# Visceral crisis in metastatic breast cancer: an old concept with new perspectives

**DOI:** 10.1016/j.clinsp.2024.100362

**Published:** 2024-05-15

**Authors:** Matheus de Oliveira Andrade, Renata Rodrigues da Cunha Colombo Bonadio, Maria Del Pilar Estevez Diz, Laura Testa

**Affiliations:** Instituto do Câncer do Estado de São Paulo (ICESP), Faculdade de Medicina da Universidade de São Paulo (FMUSP), São Paulo, Brazil

**Keywords:** Breast cancer, Neoplasm metastasis, Chemotherapy

## Abstract

Visceral Crisis (VC) in breast cancer is a critical scenario when the burden of metastatic disease results in rapid deterioration of organ functions. There are no widely accepted objective clinical criteria for the definition of VC, and different studies have reported diverse clinical conditions such as visceral crises. Diagnosis of VC is associated with a dismal prognosis and the management of this condition is currently based on limited retrospective evidence and expert opinions. International guidelines have recommended cytotoxic polychemotherapy in the management of VC, to achieve rapid symptomatic control and preserve organ function. Nevertheless, in the case of hormone receptor-positive breast cancer, the role of chemotherapy as the treatment of choice for VC has been recently questioned, since endocrine therapy plus CDK4/6 inhibitors yielded similar response rates, with better quality of life. For HER2-positive and triple-negative breast cancer, combined chemotherapy (plus HER2-directed therapy for HER2-positive) remains a standard option for VC, but novel effective drugs such as antibody-drug conjugates are emerging and their role in the VC context shall soon be elucidated. This review aims to critically discuss the definition, prognosis, management, and future directions regarding the visceral crisis in metastatic breast cancer.

## Highlights

• Visceral crisis in metastatic breast cancer is associated with a dismal prognosis.
• There is a lack of objective clinical criteria in the definition of visceral crisis.
• Visceral crisis management is currently based on limited retrospective evidence and expert opinions.
• The role of chemotherapy as the treatment of choice for visceral crisis has been recently questioned.

## Introduction

Breast cancer is the most common cancer the and main cause of cancer-related mortality in women worldwide.^1^ Despite advances in early diagnosis and curative treatments, 20 % to 30 % of patients with breast cancer develop metastatic disease, with a significant proportion having visceral involvement, either at initial presentation or as a consequence of disease progression.^2^^,^^3^

The clinical presentation of metastatic breast cancer, even in the presence of visceral involvement, is highly variable. The most critical scenario occurs when the burden of metastatic disease results in rapid deterioration of organ functions, which is considered a life-threatening condition known as Visceral Crisis (VC). Approximately 10 %‒18 % of patients with advanced breast cancer may develop visceral crises.^4^^,^^5^ Among patients with visceral metastases, a 60 % prevalence of VC has been reported.^6^ There are not widely accepted objective clinical criteria for the definition of VC, and different studies have reported diverse clinical conditions as visceral crises. Furthermore, the management of this condition is currently based on limited retrospective evidence and expert opinions since VC has been a common exclusion criterion in clinical trials. This review aims to discuss the definition, prognosis, management, and future directions regarding the visceral crisis in metastatic breast cancer.

## Methods

For the literature review, the following databases were searched: MEDLINE (PubMed), Cochrane Library, Clinical Trials, and Web of Science. The search strategy was based on the following terms: “visceral crisis” OR “visceral” OR “metastatic breast cancer” OR “advanced breast cancer”. Key publications were selected without language or date limitation (until July 2023). Titles not related to the topic were excluded before the screening. All studies addressing the topic of management of advanced breast cancer and visceral crisis were assessed. This comprehensive search strategy was conducted to minimize the risk of missing relevant literature.

### Definition of visceral crisis and clinical presentation

The definition of the term “visceral crisis” in metastatic breast cancer was initially included in guidelines in 2014 in the European School of Oncology (ESO) ‒ European Society of Medical Oncology (ESMO) 2nd International Consensus Guidelines for Advanced Breast Cancer (ABC2). In this consensus, VC is defined as severe organ dysfunction as assessed by signs and symptoms, laboratory tests, and rapid disease progression. Thus, “visceral crisis” is not the mere presence of visceral metastases, but implies a significant organic compromise, so that the most efficacious therapy may be indicated since another treatment option at progression will probably not be possible.^7^

In the ESO-ESMO Consensus Guidelines published in 2020 (ABC5), the concept of visceral crisis is maintained, and the authors specify the clinical situations in which liver and lung visceral crisis are defined:^5^

- Hepatic visceral crisis: Rapid increase in bilirubin > 1.5 times the upper limit of normality, in the absence of Gilbert's syndrome or biliary tract obstruction.
- Pulmonary visceral crisis: Rapidly increasing dyspnea at rest, not relieved by drainage of pleural effusion.

**Fig. 1:**
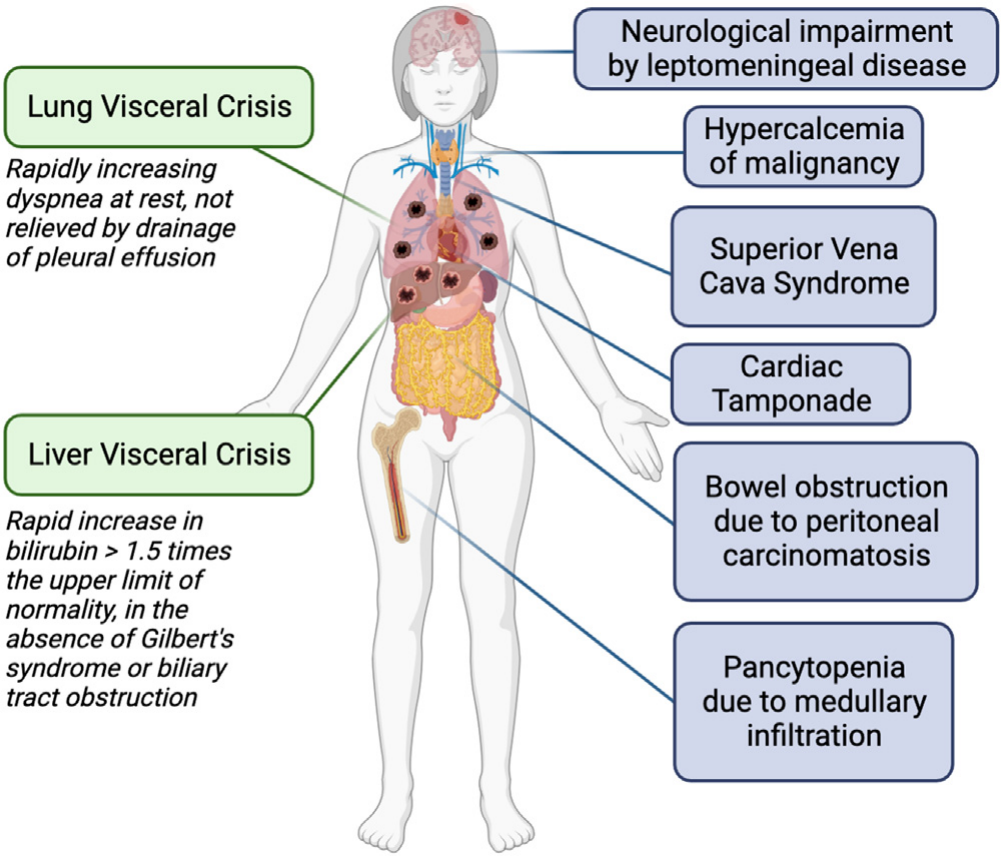
Types of visceral crisis in metastatic breast cancer. Green boxes represent the definitions found in the ABC5 Consensus. Blue boxes represent other types of visceral crisis reported in different studies. Figure created with *biorender.com*.

Although the ABC5 consensus does not define other situations of significant organic impairment in addition to liver and lung visceral crisis, other studies addressing this topic also characterize VC patients with cerebral and leptomeningeal disease with significant neurological impairment, pancytopenia due to medullary infiltration, superior vena cava syndrome, malignant bowel obstruction due to peritoneal carcinomatosis, cardiac tamponade and hypercalcemia of malignancy (Fig. 1).^4^^,^^6^^,^^8^^,^^9^

Another definition introduced in the ABC5 Consensus is the concept of “impending visceral crisis”, which is a clinical scenario where there are no criteria for visceral crisis yet, but it is foreseen to happen. An example cited in the consensus is when more than 70 % of the liver is occupied by metastases, liver transaminases are altered, but bilirubin is still normal. In this case, it is also recommended the most rapidly efficacious therapy.^5^

Several cohorts have shown diversity in the clinical presentation of visceral crisis, regarding the type of organic involvement. Sbitti et al. described 35 patients with VC, of which 55 % had liver dysfunction and 35 % respiratory failure.^8^ The higher prevalence of liver and lung involvement was also verified in another study with 261 patients in visceral crisis, with 51 % with liver failure and 17 % with pulmonary lymphangitis.^6^ In the cohort of Yang and collaborators, approximately 50 % of the patients had a visceral crisis due to liver disease, 25 % from bone marrow infiltration, 15 % from meningeal disease, and 7 % from pulmonary involvement.^4^

It is important to highlight the variability of laboratory and clinical criteria used to characterize VC in different cohorts, some of them published before ABC5 objectively defined liver and lung visceral crisis. Various studies considered increased transaminases as a criterium for liver VC even in the absence of elevated bilirubin,^6^^,^^8^^,^^9^ which would be currently defined as *impending visceral crisis* according to ABC5. Others simply considered hepatic dysfunction, without further specification, as liver VC.^4^^,^^10^ Lung visceral crisis is also a subject of controversial definition. Although most cohorts consider dyspnea due to lymphangitis or bulky lung metastases,^4^^,^^6^^,^^8^ some also include “requirement for thoracocentesis”,^9^ which is not necessarily a lung VC criterium according to the ABC5 definition.^5^

### Prognostic implications

A real-world database of 22,000 women with metastatic breast cancer reported an overall survival of 39.5 months in the whole cohort. The main negative prognostic factors were performance status, older age at diagnosis of metastases, metastasis-free interval between 6 and 24 months, presence of visceral disease, and ≥ 3 metastatic sites.^11^ A population-based analysis in the United States assessing causes of death after breast cancer diagnosis showed that approximately 50 % of deaths are related to breast cancer itself.^12^ Visceral disease is an important determinant of breast cancer mortality and accounts for 42 % of all breast cancer deaths according to a clinicopathological study.^13^

The diagnosis of visceral crisis in metastatic breast cancer is associated with poor prognosis. Sbitti et al. showed a median Overall Survival (mOS) of 4.7 weeks in a retrospective cohort of 35 patients with hormone receptor-positive and HER2-negative (HR+/HER2-) breast cancer and visceral crisis pre-treated with two hormonal therapy lines, of whom 77 % had liver metastases.^8^ Another study that included 261 patients with VC who received platinum-based chemotherapy showed a mOS of 3.7 months.^6^ Yang et al. described a longer survival of 11.2 months in a retrospective study with 133 patients, also including patients with bone marrow infiltration and meningeal involvement. In that study, overall survival ranged from 8.1 months for patients with liver dysfunction to 18 months for patients with bone marrow metastases.^4^ Thus, the type of organic involvement in patients with VC is an established prognostic factor, with hepatic involvement being more associated with worse mOS. Indeed, a review of 32 case reports of acute liver failure caused by metastases from breast cancer showed a poor prognosis, resulting in death in less than a month for most cases.^14^ On the other hand, bone marrow metastases are associated with relatively better survival rates, close to one year in some cohorts.^15^^,^^16^ It is not consensual that medullary infiltration should be considered a type of visceral crisis, due to its better prognosis. Other prognostic factors include performance status, number of previous lines of treatment, hyperbilirubinemia, increased lactate dehydrogenase, and resolution of visceral crisis after chemotherapy.^4^^,^^6^^,^^9^

### Current recommendations on the management of breast cancer visceral crisis

International guidelines recommend cytotoxic polychemotherapy in the treatment of metastatic breast cancer with visceral crisis, to achieve rapid symptomatic control and preserve organ function. Regimens with high response rates are favored in the scenario.

The NCCN (National Comprehensive Cancer Network) guideline in its fourth version of 2023, regarding systemic treatment recommendations for HR+/HER2- advanced breast cancer, states that chemotherapy combinations may be used in patients with high tumor burden, rapidly progressing disease, and visceral crisis. Other treatment regimens include PARP inhibitors in first-line therapy of BRCA1/2-mutated patients, and Antibody-Drug Conjugates (ADCs), such as trastuzumab-deruxtecan and sacituzumab-govitecan, in the second-line setting.^17^ The Objective Response Rates (ORR) observed with these first-line therapies (in trials without patients in visceral crisis) are close to 60 % with olaparib in BRCA1/2-mutated patients and with polychemotherapy.^18^^,^^19^ In posterior lines, trastuzumab-deruxtecan and sacituzumab-govitecan are associated with an ORR of 52.6 % and 21 % %, respectively.^20^^,^^21^

The ASCO (American Society of Clinical Oncology) guideline of 2021 for chemotherapy and targeted therapy for HR+/HER2- metastatic BC, although not specifically mentioning the term “visceral crisis” in its recommendations, states that chemotherapy combination regimens may be offered for “symptomatic or immediately life-threatening disease for which time may allow only one potential chance for therapy”. This recommendation extends to patients with HR+/HER2- and triple-negative without expression of Programmed cell Death Ligand-1 (PD-L1-negative) metastatic breast cancer.^22^

Such a recommendation contrasts with the therapeutic approach of some cases without symptomatic visceral disease. In metastatic luminal breast cancer without VC, for example, the standard treatment is endocrine therapy, which has comparable efficacy to chemotherapy with a better tolerability profile.^23^^,^^24^ In endocrine refractory patients, sequential single-agent chemotherapy should be offered rather than combination therapy. Metastatic triple-negative breast cancer patients without expression of PD-L1 also benefit from single-agent chemotherapy rather than polychemotherapy as first-line treatment, in a scenario without visceral crisis.^22^^,^^25^

The recommendation of cytotoxic chemotherapy in the management of visceral crisis derives from historical data indicating that, in general, chemotherapy has higher response rates than endocrine therapy, mainly when given in combination. Many of the studies from which such data were obtained included patients not screened for hormone receptor status.^26^, ^27^, ^28^ Most randomized clinical trials in metastatic breast cancer have excluded patients in VC from the study population. In addition, there is a paucity of real-world data regarding the management of breast cancer and expected response rates in the specific context of the visceral crisis. Apart from some case reports,^29^, ^30^, ^31^, ^32^ there are few retrospective cohorts, with a limited number of patients, evaluating the outcomes of VC treatment. Table 1 summarizes the key findings of the main published studies including patients with breast cancer visceral crisis.

**Table 1:** Summary of breast cancer visceral crisis cohorts.

Reference	n (patients with VC)	BC subtype	VC type	Prior palliative treatment for ABC before VC	VC Treatment regimen	Efficacy Outcomes	Prognostic factors	CT in last weeks of life
Franzoi et al. 2021^6^	261	HR+/HER2- (63.6 %)	Liver (51.3 %)	None: 12.3 %	Platinum-based CT (100 %)	mOS 3.7 months	ECOG-PS≥2	42.6 % (last 4 weeks before death)
			Lung (17.2 %)	1‒3 prior treatment lines: 44.4 %		ORR 27.2 %	Hyperbilirubinemia	
		HR+/HER2+ (6.5 %)	Brain (10.7 %)				> 3 previous treatment lines	
		HR-/HER2+ (8 %)	Peritoneum (9.5 %)	> 3 prior treatment lines: 43.3 %			Non-resolution of VC	
		TNBC (21.8 %)	Meninges (8.4 %)					
			Others (2.6 %)					
Yang et al. 2022^4^	133	HR+/HER2- (69.2 %)	Liver (50.4 %)	NR	Trastuzumab + Pertuzumab (12.78 %)	mOS 11.2 months	ECOG-PS	NR
			Bone marrow (24.8 %)				Type of VC	
		HR-/HER2+ (15 %)	Meninges (15.8 %)		Antibody–Drug Conjugate (2.26 %)	mPFS 5.2 months		
		TNBC (15.8 %)	Lung (7.5 %)		Paclitaxel (21.05 %)			
			SVCS (1.5 %)					
					Platinum (19.55 %)			
					Gemcitabine (18.79 %)			
					Eribulin (2.26 %)			
					AI (11.28 %)			
					CDK4/6 inhibitors+AI (11.28 %)			
					CDK4/6+ Fulvestrant (0.75 %)			
Funasaka et al. 2021^9^	44	HR+/HER2- (80 %)	Lung (66 %)	None: 68 %	Paclitaxel + bevacizumab (100 %)	mOS 10.6 months	ECOG-PS≥2	NR
			Liver (23 %)	CT: 1 line: 14 %; ≥2 lines: 18 %			LDH≥300	
		TNBC (20 %)	Bone marrow (16 %)			ORR 41 %	≥2nd-line CT	
				ET: NR				
			SVCS (5 %)					
Sbitti et al. 2017^8^	35	HR+/HER2- (100 %)	Liver (55 %)	2 lines of ET: 100 %	Epirubicine and cyclophosphamide (25 %)	mOS 4.7 weeks (5.8 weeks for CT; 6.2 weeks for BSC)	ECOG-PS	65 % (last 5 weeks before death)
			Lung (35 %)		Paclitaxel and bevacizumab (20 %)			
			Meninges (20 %)					
					Docetaxel (20 %)			
					BSC (34.3 %)			
Sakin et al. 2019^15^	30	HR+ (70 %)	Bone marrow (100 %)	Median of 2.5 treatment lines	Paclitaxel (60 %)	mOS 9 months	NR	NR
		HER2+ (13.3 %)			Cisplatin (13.3 %)			
					Eribulin (10 %)			
		TNBC (13.3 %)			Capecitabine (6.7 %)			
					BSC (10 %)			
Kopp et al. (2011)^16^	22	HR+ (72.7 %)	Bone marrow (100 %)	NR	Docetaxel + Doxorubicin (27.3 %)	mOS 11 months	NR	NR
		HER2 (13.6 %)						
		TNBC (9.1 %)						
					Gemcitabine + vinorelbin (22.7 %)			
					Doxorubicin liposomal, capecitabine, cyclophosphamide, docetaxel, gemcitabine, paclitaxel (4.5 %)			
Sharma et al. (2003)^33^	11	HR+ (54.5 %)	Liver (100 %)	Taxane (63.6 %)	Cisplatin + vinorelbine (100 %)	mOS 6.5 months	NR	NR
		HER2+ (36.4 %)		Anthracycline (36.4 %)		ORR 63.6 %		
		TNBC: NR		Trastuzumab (36.4 %)				
				Other (18.2 %)				

ABC, Advanced Breast Cancer; AI, Aromatase Inhibitor; BC, Breast Cancer; BSC, Best supportive Care; CDK 4/6, Cyclin-Dependent Kinase 4 and 6; CT, Chemotherapy; ET, Endocrine Therapy; HER2, Human Epidermal Growth Factor Receptor-2; HR, Hormone Receptor; mPFS, median Progression-Free Survival; mOS, median Overall Survival; NR:, Not Reported; ORR, Objective Response Rate; PS: Performance-Status; SVCS, Superior Vena Cava Syndrome; TNBC, Triple-Negative Breast Cancer; VC, Visceral Crisis.

Yang et al. examined different treatment regimens in breast cancer VC and reported a median overall survival of 6.2 months with chemotherapy, 13.2 months in the anti-Her2 therapy group, and 24.3 months in the Endocrine Therapy (ET) group. Dose reduction was needed in 31.7 % of patients treated with chemotherapy, 25 % in the anti-Her2 therapy group, and 16 % in the ET group. The authors argued that ET exhibited good safety and efficacy in HR+/HER2- patients. Nevertheless, it is not specified in which type of visceral crisis ET was applied, and there was a selection bias to patients with better prognosis, such as bone marrow metastasis, and good performance scores. Indeed, ECOG-PS (Eastern Cooperative Oncology Group Performance Status) and type of visceral crisis were prognostic factors established in this cohort.^4^

The main guidelines do not suggest specific Chemotherapy (CT) regimens in breast cancer VC. Previous studies assessed different CT regimens and reported diverse efficacy and toxicity outcomes. Franzoi et al. described an Objective Response Rate (ORR) of 27 % with platinum-based chemotherapy in VC and a mOS of 3.7 months. According to the authors, platinum-based chemotherapy use may be justified by not requiring dose adjustments based on liver function, allowing treatment with full-dose chemotherapy in a clinical scenario when maximum response is needed.^6^ Another platinum-based regimen studied was cisplatin and vinorelbine, in a pilot study with eleven patients affected by liver visceral crisis. This treatment was associated with an ORR of 63.6 % and mOS of 6.5 months. Myelosuppression was the most frequently reported adverse event, and there was a treatment-related death due to intracerebral hemorrhage.^33^

In situations in which liver function is preserved, other chemotherapy combinations with known efficacy in breast cancer are valuable options, such as AC (anthracycline and cyclophosphamide) or taxane-based combinations, being associated with response rates around 47 % to 57 % in the first-line setting outside the context of VC.^19^^,^^34^ Paclitaxel combined with bevacizumab is another regimen previously studied in VC, with a mOS of 10.6 months and ORR of 41 %. In this cohort, 30 % of patients discontinued treatment because of adverse events.^9^ Paclitaxel was also a regimen widely used in a cohort of VC due to bone marrow metastasis, in which 60 % received paclitaxel as first-line treatment, with a mOS of 9 months.^15^

Comparisons of efficacy outcomes between these cohorts must be avoided, considering different baseline patients’ characteristics. The number of previous treatment lines is an important prognostic factor, and it is expected a worse prognosis in cohorts with more prior treatment lines before therapy for VC. For example, Funasaka et al. described 10.6 months of mOS in a population in which 68 % of patients had received paclitaxel and bevacizumab as a first-line therapy for VC.^9^ On the other hand, Franzoi's cohort reported a mOS of 3.7 months in a population with only 12.3 % of patients receiving treatment for VC in a first-line scenario.^6^ Sbitti et al. selected patients regardless of cancer therapy, including 34.3 % of the sample submitted only to supportive care (BSC), with a mOS of 5.8 weeks for chemotherapy and 6.2 weeks for BSC.^8^

Despite the retrospective nature of most available studies evaluating outcomes in patients with visceral crisis, the cohorts suggest a poor prognosis for patients treated with chemotherapy. Many patients with VC develop significant deterioration in performance status and organic dysfunction that affect the tolerability of most chemotherapeutic agents. It is possible that traditional chemotherapy could reduce the quality of life without significantly improving survival.^35^ A high prevalence of patients receiving chemotherapy in the last month of life is reported, estimated from 42 % to 65 % in different cohorts.^6^^,^^8^ This is an important consideration since not in all situations the presence of VC will justify the use of active oncologic therapy. In some situations, especially those of previously treated patients, and when performance status is poor, the treatment options available might not be associated with considerable response rates, and treatment risks can overcome its potential benefit, or it can represent a futile strategy.

Finally, the need for combined polychemotherapy as the choice of treatment has been recently questioned for VC in hormone-receptor-positive HER2-negative breast cancer, as discussed in the next section of this review. Also, facing the emerging new oncologic therapies, for the first time, the ESO-ESMO ABC guideline (2020) recommends for the treatment of VC the “most rapidly efficacious therapy, which is not necessarily chemotherapy in all situations”.^5^ In this context, Cyclin-Dependent Kinase 4 and 6 (CDK4/6) inhibitors, Poly (ADP-ribose) Polymerase (PARP) inhibitors, immune checkpoint inhibitors, and Antibody-Drug Conjugates (ADCs) could emerge as potential therapeutic options in the visceral crisis setting.

### Perspectives

Recent studies have suggested that the use of CDK4/6 inhibitors has increased since their approval, reaching over 55 % of HR+/HER2- patients as first-line therapy, in countries where this treatment is accessible.^36^ CDK4/6 inhibitors act through inhibition of cyclin D-CDK4/6 complex activation, thus preventing the G1/S transition. Their mechanism of action halting the cell cycle is somehow similar to many traditional chemotherapeutic agents, although the combination of CDK4/6 inhibitors with endocrine therapy has provided better results than the combination of ET and chemotherapy in HR+/HER2- breast cancer.^24^^,^^37^ The combination of endocrine therapy and CDK4/6 inhibitors is associated with response rates close to 50 % in patients with luminal breast cancer and visceral metastases, but visceral crisis was an explicit exclusion criterion in the pivotal trials.^38^, ^39^, ^40^, ^41^

Previous exploratory data with abemaciclib and endocrine therapy showed that patients with poor prognostic features, such as liver metastasis, derived more benefit from this therapy,^42^ which endorses the rationale of assessing this combination in breast cancer VC. Recent preliminary real-world retrospective data suggest that the combination of endocrine therapy with CDK4/6 inhibitors has response rates and time to response comparable to chemotherapy, with a better tolerability profile, in the scenario of visceral crisis. Dawood et al. presented at the 2021 ASCO conference the results of a retrospective analysis in which patients with luminal breast cancer and VC treated with CDK4/6 inhibitors had a 5-month improvement in overall survival compared to chemotherapy (11 vs. 6 months).^10^^,^^26^

The first prospective trial evaluating the use of endocrine therapy and CDK4/6 inhibitor combination in visceral crisis, the RIGHT Choice trial, was presented at the San Antonio Breast Cancer Symposium of 2022. This randomized phase II trial compared ribociclib and ET versus physician's choice combination chemotherapy in premenopausal or perimenopausal women with aggressive HR+/HER2- advanced breast cancer. Their criteria for aggressive disease included symptomatic visceral metastases, rapid disease progression or impending visceral compromise, and markedly symptomatic nonvisceral disease. First-line ribociclib and ET demonstrated a statistically significant PFS benefit in comparison to combination CT (24.0 vs. 12.3 months; HR = 0.54). It also showed a similar median time to onset of response (4.9 vs. 3.2 months) and objective response rate (65.2 % vs. 60 %), with fewer treatment-related adverse events.^43^

It is relevant to mention that half of the study population comprised patients in visceral crisis (according to ABC3 and NCCN guidelines, available at the time of study design). In addition, total bilirubin ≤1.5 ULN was a mandatory inclusion criterion, thus excluding patients with liver VC according to ABC5 criteria. Furthermore, in the subgroup analysis, the PFS benefit was not seen in the VC patients. These particularities limit the external validity of the results in the strict visceral crisis scenario, regarding patients in hepatic VC, which are known to have a worse prognosis.

There are two ongoing phase II trials assessing the combination of endocrine therapy and CDK4/6 inhibitors (abemaciclib and dalpiciclib) in HR+/HER2- breast cancer visceral crisis first-line therapy (Table 2). Such clinical trials have broader inclusion criteria, also assessing patients with hyperbilirubinemia in the context of liver VC. Nevertheless, both trials include patients with dyspnea from pleural effusion, which is not characterized *per se* as a visceral crisis according to ABC5.

**Table 2:** Ongoing trials in metastatic breast cancer visceral crisis.

Clinical Trials ID	Design	Population	Intervention	Primary Outcome	Main Secondary Outcomes	Status
NCT04681768	Single-arm phase II	HR+/HER2- ABC with symptomatic visceral metastases or high tumor burden	Abemaciclib + ET (1st line)	ORR	DCR, DoR, PFS, TTF, AE, PRO	Recruiting
NCT05431504	Single-arm phase II	HR+/HER2- ABC with VC	Dalpiciclib + ET (1st line)	6-month OS	OS, PFS, TTF, ORR, CBR, DoR, TTR, AE	Not yet recruiting

ABC, Advanced Breast Cancer; AE, aAdverse Events; DCR, Disease Control Rate; DoR, Duration of Response; ET, Endocrine Therapy; HER2, Human Epidermal growth factor Receptor-2; HR, Hormone Receptor; PFS, Progression-Free Survival; PRO, Patient-Reported Outcomes; OS, Overall Survival; ORR, Objective Response Rate; TTF, Time to Treatment Failure; VC, Visceral Crisis.

The ongoing trials in metastatic breast cancer VC are focused on HR+/HER2- patients. Despite the advances in the treatment of HER2-positive/low TNBC with ADCs and immune checkpoint inhibitors, there is a lack of data on these therapies’ efficacy and safety in the visceral crisis scenario, since VC patients are not well represented in the studies.^20^^,^^44^^,^^45^ Current recommendations of these drugs in advanced breast cancer are extrapolated to patients in VC considering the principle of treating them with the most rapidly efficacious therapy that is available. However, questions regarding the safety of these agents still remain. For example, pulmonary toxicity of trastuzumab-deruxtecan could be a concern in HER2-positive/low patients in lung visceral crisis.

For these perspectives to turn into clinical practice, trials including patients with visceral crisis are needed, to better assess the efficacy and safety of potential therapies in this life-threatening scenario.

## Conclusions

Visceral crisis in metastatic breast cancer is a challenging situation from its definition to its management. There is still a lack of objective clinical criteria and a gap between the ABC5 definition (limited to lung and liver VC examples) and other studies with broader conditions defined as VC (bone marrow, meningeal, peritoneal, central nervous system). The variability in the definition of visceral crisis and patients’ characteristics among different cohorts contributes to diverse results regarding efficacy outcomes, although it is consensual that the overall survival is poor.

The general guideline recommendation of treatment with the most rapidly efficacious therapy must be put in perspective considering the frailty of patients in visceral crisis and other prognostic factors, such as the number of prior treatment lines. Prognostication in advanced breast cancer still often relies on subjective clinical judgment. More accurate and objective prognostic tools could allow the best decisions on the management of the visceral crisis, considering its dismal prognosis. Taking these factors into account could help physicians to better differentiate patients who are likely to benefit from oncologic treatment from those for whom such therapy would be futile or potentially harmful.

## Funding

This research did not receive any specific grant from funding agencies in the public, commercial, or not-for-profit sectors.

## Declaration of competing interest

The authors declare no conflicts of interest.
